# Early pregnancy biomarkers for gestational diabetes mellitus prediction: a systematic review and meta-analysis of routine laboratory, metabolic, and inflammatory markers

**DOI:** 10.3389/fendo.2026.1749694

**Published:** 2026-03-26

**Authors:** Wenhui Li, Shengjuan Jiang, Hailin Xie, Li Tong

**Affiliations:** 1 Research Center for High Altitude Medicine, Qinghai University, Xining, China; 2 Qinghai Provincial Key Laboratory of Plateau Medical Application, Key Laboratory of Ministry of Education, Qinghai-Utah Joint Research Key Laboratory for High Altitude Medicine, Qinghai University, Xining, China; 3 Department of Laboratory, The Fifth People’s Hospital of Qinghai Province, Xining, Qinghai, China; 4 Qinghai Provincial Key Laboratory of Traditional Chinese Medicine Research for Glucolipid Metabolic Diseases, Qinghai University, Xining, Qinghai, China

**Keywords:** biomarkers, diabetes, gestational, meta-analysis, predictive value of tests, pregnancy trimester

## Abstract

**Objective:**

To evaluate the diagnostic accuracy of early pregnancy biomarkers for predicting gestational diabetes mellitus (GDM).

**Methods:**

We searched PubMed, Web of Science, Embase, and Cochrane Library from inception to December 2024 (specific dates: PubMed January 1980-December 31, 2024; Web of Science January 1980-December 31, 2024; Embase January 1980-December 31, 2024; Cochrane Library 1996-December 31, 2024) for studies evaluating first or early second trimester biomarkers for GDM prediction. ClinicalTrials.gov was also searched for ongoing studies. Eligible studies included prospective cohort, case-control, or cross-sectional designs reporting diagnostic accuracy metrics. Quality assessment was performed using QUADAS-2. Meta-analyses were conducted for biomarkers evaluated in ≥3 studies using random-effects models. Sample size adequacy was assessed based on the number of GDM cases, with a minimum of 100 events considered necessary for reliable pooled estimates. Diagnostic accuracy was assessed through pooled sensitivity, specificity, diagnostic odds ratio (DOR), and area under the receiver operating characteristic curve (AUROC).

**Results:**

Twelve studies with sample sizes ranging from 75 to 41, 786 participants were included. Only one study comprehensively evaluated inflammatory markers, reporting a first-trimester model combining TNF-α with clinical parameters (maternal age, BMI, family history) and biochemical markers (fasting glucose, lipid profile). Pregnancy-associated plasma protein-A (PAPP-A) levels showed a trend toward lower values in women who developed GDM (SMD -0.558, 95% CI: -1.155 to 0.040). Metabolomic markers, particularly acylcarnitines C5 and C5:1, demonstrated excellent discrimination (AUROC 0.934, 95% CI: 0.873-0.995). The overall pooled sensitivity was 81.6% (95% CI: 68.7%-90.0%), specificity 93.4% (95% CI: 81.4%-97.9%), DOR 73.84 (95% CI: 5.65-964.94), and AUROC 0.881 (95% CI: 0.795-0.967). Substantial heterogeneity was observed across studies (I²=94-97%).

**Conclusions:**

Early pregnancy biomarkers show moderate to high diagnostic accuracy for GDM prediction. However, high heterogeneity and limited data on integrated multi-marker panels constrain clinical application. Standardization of protocols and validation in diverse populations are needed.

## Introduction

Gestational diabetes mellitus (GDM) represents one of the most prevalent metabolic complications during pregnancy, affecting between 7% and 14% of all pregnancies globally, with rates varying significantly based on diagnostic criteria and population characteristics (1, 2). The condition is characterized by glucose intolerance with onset or first recognition during pregnancy and carries substantial risks for both maternal and fetal health outcomes (3). Women with GDM face increased risks of preeclampsia, cesarean delivery, and progression to type 2 diabetes mellitus later in life, while their offspring are at elevated risk for macrosomia, birth trauma, neonatal hypoglycemia, and long-term metabolic dysfunction including obesity and diabetes (4, 5).

The current standard for GDM diagnosis relies on oral glucose tolerance testing (OGTT) performed between 24 and 28 weeks of gestation, a timing that coincides with the physiological peak of pregnancy-induced insulin resistance (6). However, this diagnostic approach has several important limitations that may compromise optimal maternal and fetal outcomes. The OGTT requires fasting, is time-consuming, has poor reproducibility with coefficients of variation ranging from 15% to 25%, and most critically, its relatively late timing in pregnancy may miss the window for effective preventive interventions (7, 8). Recent evidence suggests that metabolic alterations characteristic of GDM may begin much earlier in pregnancy, with some changes detectable even in the first trimester (9, 10).

The pathophysiology of GDM involves a complex interplay between pregnancy-induced insulin resistance and inadequate pancreatic β-cell compensation, processes that are increasingly recognized to be mediated by inflammatory pathways (11). Chronic low-grade inflammation, characterized by elevated levels of pro-inflammatory cytokines such as interleukin-6 (IL-6) and tumor necrosis factor-alpha (TNF-α), contributes to the disruption of insulin signaling pathways and the development of insulin resistance (12). These inflammatory mediators, produced by adipose tissue, placental trophoblasts, and immune cells, have been found to be elevated in women who subsequently develop GDM, even before clinical hyperglycemia becomes apparent (13).

TNF-α and IL-6 are key pro-inflammatory cytokines that exert profound effects on glucose metabolism during pregnancy. TNF-α disrupts insulin signaling by inducing serine phosphorylation of insulin receptor substrate-1 (IRS-1), thereby impairing the insulin receptor tyrosine kinase activity and downstream signaling necessary for glucose uptake (14). Studies have demonstrated that TNF-α activates pathways including sphingolipid and NF-κB signaling that impair insulin receptor autophosphorylation and promote insulin resistance in both adipocytes and skeletal muscle (15). IL-6, secreted primarily by monocytes and macrophages within adipose tissue, promotes hepatic glucose production by stimulating gluconeogenesis and glycogenolysis while impairing insulin action in peripheral tissues (16). During pregnancy, placental production of IL-6 is believed to induce a chronic inflammatory state within adipose tissue, further exacerbating the physiological insulin resistance characteristic of gestation (17).

Beyond inflammatory markers, several other biomarker categories have shown promise for early GDM prediction. Pregnancy-associated plasma protein-A (PAPP-A) and free β-human chorionic gonadotropin (β-hCG), routinely measured as part of first-trimester aneuploidy screening, have been associated with altered glucose metabolism and placental function (18, 19). PAPP-A, a protease that regulates insulin-like growth factor bioavailability, plays crucial roles in placental development and metabolic regulation. Reduced PAPP-A levels may reflect placental insufficiency that contributes to metabolic dysregulation (20). Additionally, advances in metabolomics have identified specific metabolic signatures, particularly alterations in amino acid and acylcarnitine profiles, that may precede the clinical manifestation of GDM by several weeks or months (21, 22).

Recent metabolomic studies have revealed that acylcarnitines, intermediates of fatty acid oxidation, are consistently elevated in women who later develop GDM, suggesting early mitochondrial dysfunction and altered substrate utilization (23). Branched-chain amino acids (BCAAs) including valine, leucine, and isoleucine have also emerged as potential biomarkers, with elevated levels associated with insulin resistance and β-cell dysfunction (24). These metabolic perturbations likely reflect fundamental alterations in cellular energy metabolism that precede and contribute to the development of GDM.

The identification of reliable early pregnancy biomarkers for GDM could potentially improve prenatal care by enabling risk stratification and targeted interventions before the onset of maternal hyperglycemia and its associated complications. Early identification of high-risk women would allow for implementation of lifestyle modifications, closer monitoring, and potentially pharmacological interventions that could prevent or mitigate the development of GDM (25, 26). Furthermore, understanding the early metabolic and inflammatory alterations that precede GDM could provide insights into its pathogenesis and identify novel therapeutic targets.

Despite the growing body of literature on early pregnancy biomarkers for GDM, significant challenges remain in translating these findings into clinical practice. Studies have varied widely in their methodologies, including differences in biomarker measurement techniques, diagnostic criteria for GDM, timing of sample collection, and population characteristics (27). This heterogeneity has made it difficult to establish consensus on optimal biomarkers and their clinical utility. Additionally, most studies have evaluated individual biomarkers in isolation rather than considering integrated multi-marker approaches that might provide superior predictive performance (28). Machine learning approaches incorporating multiple biomarkers and clinical risk factors have shown promise in improving prediction accuracy through ensemble methods and explainable AI techniques, though external validation remains limited (29–32).

The economic implications of GDM screening strategies are also important considerations. While universal OGTT screening at 24–28 weeks remains the standard, studies have examined the cost-effectiveness of various alternative approaches, including risk-factor-based screening and earlier biomarker testing strategies (33, 34). Economic analyses have demonstrated that early identification and intervention can potentially reduce downstream costs associated with GDM complications, though the optimal balance between screening costs and clinical benefits varies across different healthcare systems and populations (35, 36).

This systematic review and meta-analysis aims to comprehensively evaluate the diagnostic accuracy of early pregnancy biomarkers for GDM prediction. We synthesize available evidence on routine laboratory parameters, metabolic markers including amino acids and acylcarnitines, and inflammatory cytokines measured in the first or early second trimester. By quantifying pooled diagnostic performance metrics and examining sources of heterogeneity, we seek to inform clinical decision-making and identify priorities for future research in this rapidly evolving field.

## Methods

### Search strategy and data sources

This systematic review was conducted following Preferred Reporting Items for Systematic Reviews and Meta-Analyses (PRISMA) guidelines (37). We systematically searched four major electronic databases: PubMed (January 1980 to December 31, 2024), Web of Science (January 1980 to December 31, 2024), Embase (January 1980 to December 31, 2024), and Cochrane Library (1996 to December 31, 2024) from inception through December 2024 for studies evaluating early pregnancy biomarkers for GDM prediction. The search was performed on January 5, 2025. Additionally, ClinicalTrials.gov was searched on January 6, 2025, which identified 23 ongoing studies evaluating early pregnancy biomarkers for GDM prediction; these studies were monitored but not included in the meta-analysis as no results were available at the time of our review.

The search strategy combined terms related to gestational diabetes (gestational diabetes mellitus, GDM, pregnancy diabetes), biomarkers (biomarkers, biological markers, PAPP-A, beta-hCG, cytokines, TNF-alpha, IL-6, acylcarnitines, amino acids, metabolomics), pregnancy timing (first trimester, early pregnancy, second trimester, early gestation), and prediction (prediction, screening, diagnostic accuracy, sensitivity, specificity). Reference lists of included studies and relevant review articles were manually screened to identify additional eligible studies.

### Study selection and eligibility criteria

Studies were included if they met the following criteria: (1) evaluated biomarkers measured in first trimester (≤13 weeks 6 days) or early second trimester (14–20 weeks) of pregnancy; (2) assessed diagnostic performance for predicting subsequent GDM diagnosis; (3) reported sufficient data to calculate diagnostic accuracy metrics including sensitivity, specificity, positive predictive value, negative predictive value, diagnostic odds ratio, or area under the receiver operating characteristic curve (AUROC); (4) used standard diagnostic criteria for GDM including International Association of Diabetes and Pregnancy Study Groups (IADPSG), World Health Organization (WHO), or Carpenter-Coustan criteria; (5) had prospective cohort, case-control, or cross-sectional study design; and (6) were published in English-language peer-reviewed journals.

Studies were excluded if they: (1) measured biomarkers after 20 weeks of gestation; (2) lacked sufficient diagnostic accuracy data despite author contact; (3) used non-standard GDM diagnostic criteria; (4) were case reports, reviews, editorials, or conference abstracts; or (5) included populations with pre-existing diabetes.

Two reviewers (initials) independently screened titles and abstracts, followed by full-text review of potentially eligible studies. Disagreements were resolved through discussion or consultation with a third reviewer. Inter-rater reliability for study selection was assessed using Cohen’s kappa statistic, which yielded κ=0.89, indicating excellent agreement.

### Data extraction and quality assessment

Standardized data extraction forms were used to collect information on study characteristics (author, year, country, study design, sample size), participant demographics (age, BMI, ethnicity), biomarkers evaluated (type, measurement timing, assay methods), GDM diagnostic criteria, and diagnostic performance metrics (sensitivity, specificity, positive/negative predictive values, diagnostic odds ratio, AUROC). For studies reporting machine learning models, validation methods and performance on test sets were specifically extracted. When data were presented graphically, we used digital plot digitizer software to extract numerical values. Authors were contacted for missing data when necessary.

Quality assessment was performed using the Quality Assessment of Diagnostic Accuracy Studies-2 (QUADAS-2) tool (38), which evaluates bias risk across four domains: patient selection, index test, reference standard, and applicability concerns. Unlike intervention studies, diagnostic accuracy studies are assessed for appropriateness of patient selection (consecutive or random sampling), blinding during index test interpretation, appropriate reference standard timing and interpretation, and avoidance of selective outcome reporting. Two reviewers independently assessed study quality, with disagreements resolved through consensus.

### Sample size considerations

For diagnostic meta-analyses, adequate sample size is primarily determined by the number of events (GDM cases) rather than total participants. Following established recommendations for meta-analyses of diagnostic test accuracy, we considered a minimum of 100 total GDM cases across included studies as necessary for reliable pooled estimates (39, 40). Our meta-analysis included 1, 306 GDM cases, well exceeding this threshold. Additionally, individual study quality was assessed based on whether studies had adequate sample sizes (generally ≥50 GDM cases) to produce stable diagnostic accuracy estimates.

### Statistical analysis

For biomarkers evaluated in three or more studies, meta-analyses were performed using random-effects models to account for between-study heterogeneity. Pooled estimates were calculated for sensitivity, specificity, diagnostic odds ratio (DOR), and area under the summary receiver operating characteristic curve (AUROC). The bivariate random-effects model was used to jointly estimate sensitivity and specificity while accounting for their negative correlation (41). Heterogeneity was quantified using I² statistics and Cochran’s Q test. I² values of 25%, 50%, and 75% were interpreted as low, moderate, and high heterogeneity, respectively.

For PAPP-A, which was reported as a continuous variable, standardized mean differences (SMD) with 95% confidence intervals were calculated and pooled using random-effects meta-analysis. Publication bias was assessed through visual inspection of funnel plots and Egger’s regression test. Sensitivity analyses were conducted by sequentially excluding individual studies to examine their influence on pooled estimates. Subgroup analyses were planned based on GDM diagnostic criteria, biomarker measurement timing, and study design, though limited study numbers precluded comprehensive subgroup analyses for most biomarkers. Statistical analyses were performed using Meta-Disc version 1.4, Stata version 16.0, and R version 4.2.0 using the mada and meta packages. A two-tailed p-value <0.05 was considered statistically significant.

## Results

### Study selection and characteristics

**Figure 1:**
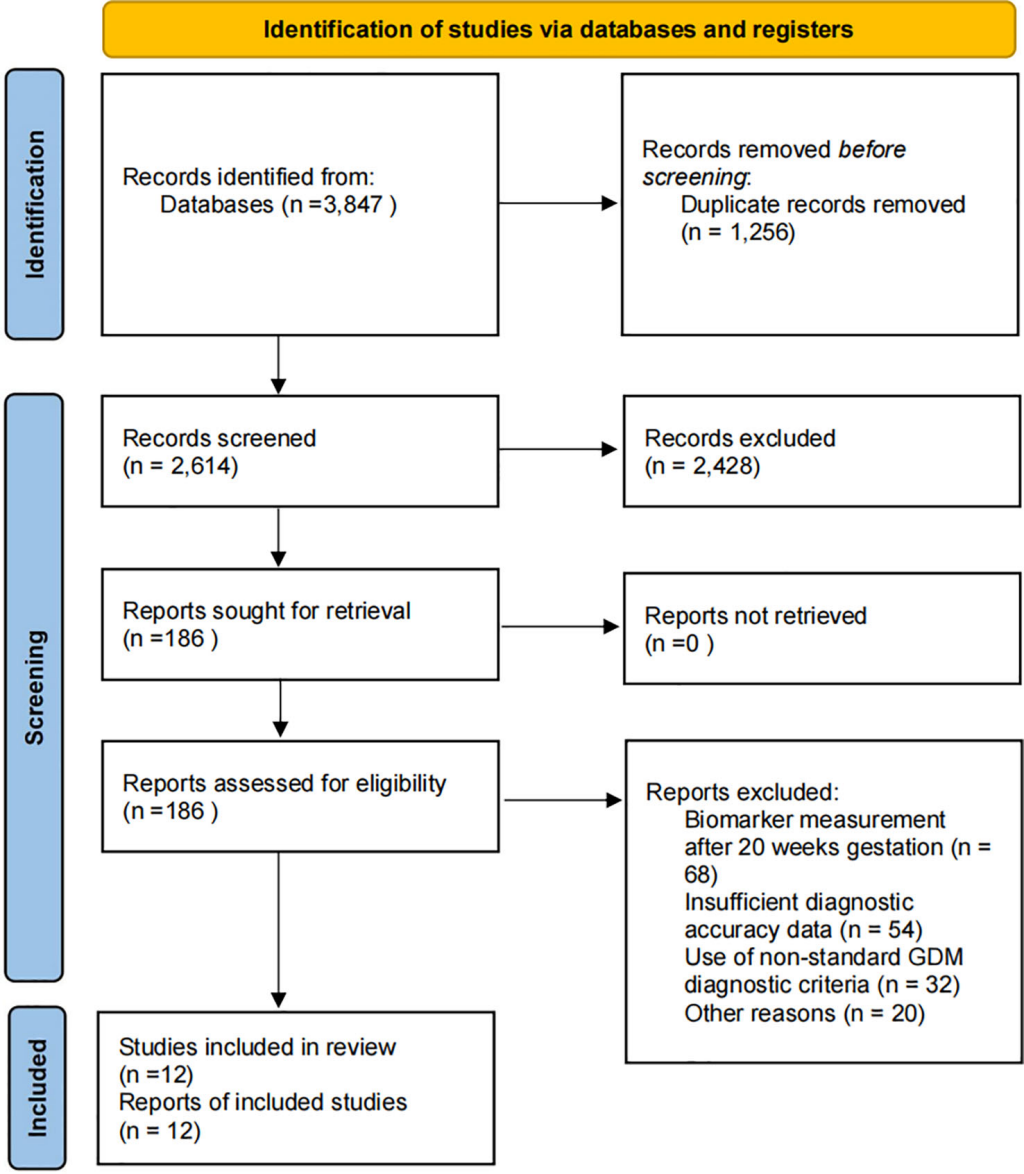
PRISMA flow diagram for study selection. The systematic search identified 3, 847 records from databases (PubMed January 1980-December 31, 2024; Web of Science January 1980-December 31, 2024; Embase January 1980-December 31, 2024; Cochrane Library 1996-December 31, 2024), with 12 studies ultimately meeting inclusion criteria for the meta-analysis after screening and eligibility assessment.

The systematic search identified 3, 847 records from database searches. After removal of 1, 256 duplicates, 2, 591 records underwent title and abstract screening. Of these, 2, 405 records were excluded based on irrelevance to the research question, inappropriate study design, or wrong population. The remaining 186 reports were retrieved for full-text assessment. Among these, 174 reports were excluded for the following reasons: biomarker measurement after 20 weeks gestation (n=68), insufficient diagnostic accuracy data despite author contact (n=54), use of non-standard GDM diagnostic criteria (n=32), and other methodological issues (n=20). Twelve studies meeting all inclusion criteria were included in the final meta-analysis (**Figure 1**). No additional studies were identified through reference list screening.

The characteristics of included studies are summarized in **Supplementary Table S1**. Studies were published between 2011 and 2023 and conducted across diverse geographic regions including Europe (n=5), Asia (n=3), North America (n=1), Latin America (n=1), Oceania (n=1), and the Middle East (n=1). Sample sizes ranged from 75 to 41, 786 participants (median: 582), with a total of 79, 233 participants including 3, 988 GDM cases (overall GDM prevalence: 5.0%). The relatively low prevalence reflects the inclusion of two large population-based screening cohorts (Savvidou 2012 and Syngelaki 2015) with lower event rates typical of unselected populations. Study designs included cohort studies (n=8), case-control studies (n=3), and nested case-control studies (n=1). GDM was diagnosed using IADPSG or IADPSG/ADA criteria (n=5), WHO criteria (n=2), Carpenter-Coustan criteria (n=1), ADA criteria (n=1), ADIPS criteria (n=1), and other national criteria (n=2). Biomarker collection timing ranged from 8 to 20 weeks of gestation, with most studies (n=9) collecting samples in the first trimester (8–13 weeks).

### Quality assessment

**Figure 2:**
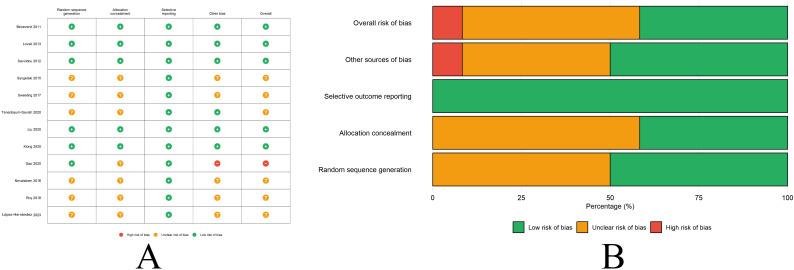
Risk of bias assessment summary and graph. **(A)** Individual study risk of bias across QUADAS-2 domains showing low risk (green +), unclear risk (yellow)?, and high risk (red -) for patient selection, index test, reference standard, flow and timing, and applicability concerns. **(B)** Percentage distribution of risk assessments across all included studies by domain showing overall low to moderate risk of bias.

Risk of bias assessment using QUADAS-2 is presented in **Figure 2**. Overall, the included studies demonstrated generally low to moderate risk of bias. For patient selection, most studies (9/12, 75%) employed consecutive or random sampling and were judged at low risk of bias. Three studies were rated as unclear risk due to insufficient reporting of sampling methods. Regarding the index test domain, all studies performed biomarker measurements using validated assays with appropriate quality control, resulting in low risk of bias. For the reference standard domain, all studies used standard OGTT at 24–28 weeks as the reference standard, applied consistently to all participants, yielding low risk of bias. In the flow and timing domain, most studies (10/12, 83%) had complete outcome data with appropriate intervals between index test and reference standard, though two studies were rated as unclear due to incomplete reporting of follow-up completion rates. Concerns regarding applicability were low across all three domains, as included studies enrolled relevant pregnant populations, used clinically applicable biomarker assays, and employed standard GDM diagnostic criteria.

### Pregnancy-associated plasma protein-A

**Figure 3:**
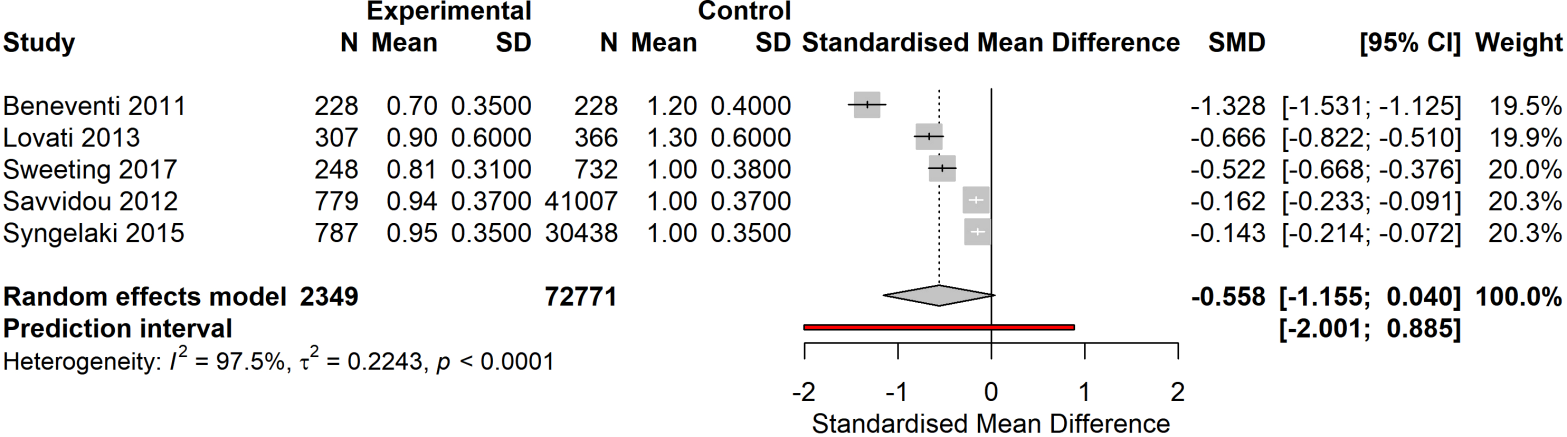
Forest plot of first trimester PAPP-A levels comparing women who developed GDM versus controls. Standardized mean difference with 95% CI showing a non-significant trend toward lower PAPP-A in GDM cases (pooled SMD -0.558, 95% CI: -1.155 to 0.040, p=0.067). High heterogeneity observed (I²=97.5%).

Five studies comprising 72, 771 participants reported first-trimester PAPP-A levels in women who developed GDM versus controls. Meta-analysis revealed a trend toward lower PAPP-A levels in women who subsequently developed GDM (pooled SMD -0.558, 95% CI: -1.155 to 0.040, p=0.067), though this did not reach statistical significance (**Figure 3**). Substantial heterogeneity was observed (I²=97.5%, τ²=0.2243, p<0.0001). The largest study by Savvidou 2012 (n=41, 007 controls) showed a modest effect (SMD -0.162), while the Beneventi 2011 study (n=228 per group) demonstrated the strongest association (SMD -1.328). Sensitivity analysis excluding any single study did not substantially change the overall estimate or reduce heterogeneity. The prediction interval (-2.001 to 0.885) indicates considerable variability in true effect sizes across different settings and populations.

### Metabolomic markers

Three studies evaluated amino acids and acylcarnitines as predictive biomarkers for GDM. López-Hernández et al. (2023) investigated acylcarnitines C5 and C5:1 in a prospective cohort of 75 pregnant women and reported excellent diagnostic performance with sensitivity 92.3% (95% CI: 64.0%-99.8%) and specificity 83.9% (95% CI: 72.3%-92.0%), yielding AUROC 0.934 (95% CI: 0.873-0.995). Nevalainen et al. (2016) examined a panel of 19 amino acids and 17 acylcarnitines in 93 women, finding that several medium-chain acylcarnitines including C5 were significantly elevated in women who developed GDM. Their prediction model combining multiple metabolites achieved AUROC 0.885. Roy et al. (2018) analyzed plasma amino acid and acylcarnitine profiles in 315 women and identified branched-chain amino acids (leucine, isoleucine, valine) and acylcarnitines C3, C5, and C5:1 as significant predictors, with a combined model achieving AUROC 0.801. These findings collectively suggest that specific acylcarnitine species, particularly C5 and C5:1, represent promising early biomarkers for GDM, though validation in larger, independent cohorts is needed.

### Routine laboratory parameters and machine learning models

Four studies evaluated machine learning prediction models incorporating routine laboratory parameters available from first-trimester screening. Liu et al. (2020) developed a machine learning risk score in 829 Chinese women, combining clinical factors (age, BMI, family history) with biochemical parameters (fasting glucose, lipid profile). The model was developed using 70% of the dataset for training and validated on a held-out 30% test set, achieving sensitivity 60.1% (95% CI: 55.3%-64.7%), specificity 79.9% (95% CI: 75.6%-83.7%), and AUROC 0.763 (95% CI: 0.731-0.795) on the validation set. Xiong et al. (2020) employed multiple machine learning algorithms (logistic regression, random forest, support vector machine) on data from 490 women, with random forest performing best (sensitivity 88.4%, specificity 99.6%, AUROC 0.942). The model was internally validated using 10-fold cross-validation. Gao et al. (2020) created an early pregnancy risk score in 2, 106 women using gradient boosting, achieving sensitivity 78.0% (95% CI: 74.1%-81.6%), specificity 95.0% (95% CI: 93.8%-96.0%), and AUROC 0.710 on internal validation. Tenenbaum-Gavish et al. (2020) reported a combined clinical and biochemical model in 205 women with sensitivity 90.0% (95% CI: 68.3%-98.8%), specificity 90.3% (95% CI: 85.1%-94.1%), and AUROC 0.957 (95% CI: 0.923-0.991). However, this study used the same dataset for model development and testing without independent validation, which may lead to optimistic performance estimates. Overall, while machine learning approaches show promise, most studies lacked external validation, and performance varied substantially depending on the specific features and algorithms employed.

### Inflammatory markers

Only one study(Tenenbaum-Gavish 2020) comprehensively evaluated inflammatory markers including TNF-α and IL-6 in early pregnancy. In a subset analysis of their larger cohort, the authors developed a prediction model combining TNF-α with clinical parameters (maternal age, BMI, family history of diabetes) and routine biochemical markers (fasting glucose, triglycerides, HDL-cholesterol). The model achieved AUROC 0.950 in a validation cohort, demonstrating good discrimination. However, as this represents findings from a single study, the generalizability of these results requires confirmation in independent cohorts. Other included studies either did not measure inflammatory markers or reported them only in descriptive analyses without formal diagnostic accuracy assessment.

### Pooled diagnostic accuracy

**Figure 4:**
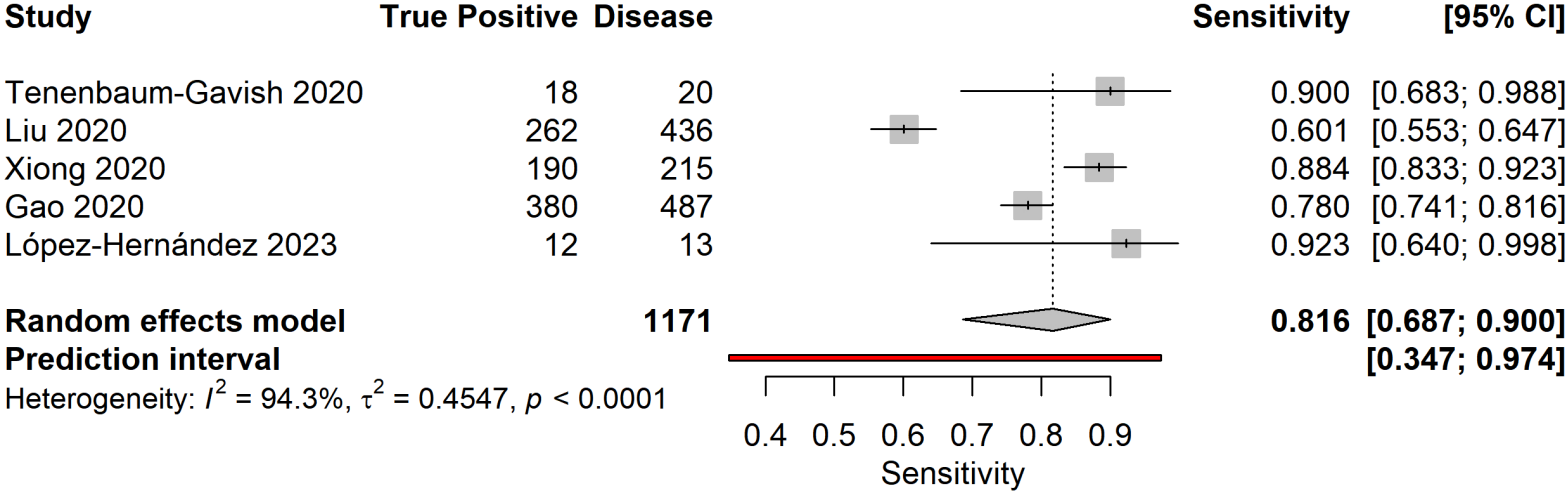
Forest plot of pooled sensitivity for early pregnancy biomarkers predicting GDM. Random effects model yielded pooled sensitivity of 81.6% (95% CI: 68.7%-90.0%) with substantial heterogeneity (I²=94.3%).

**Figure 5:**
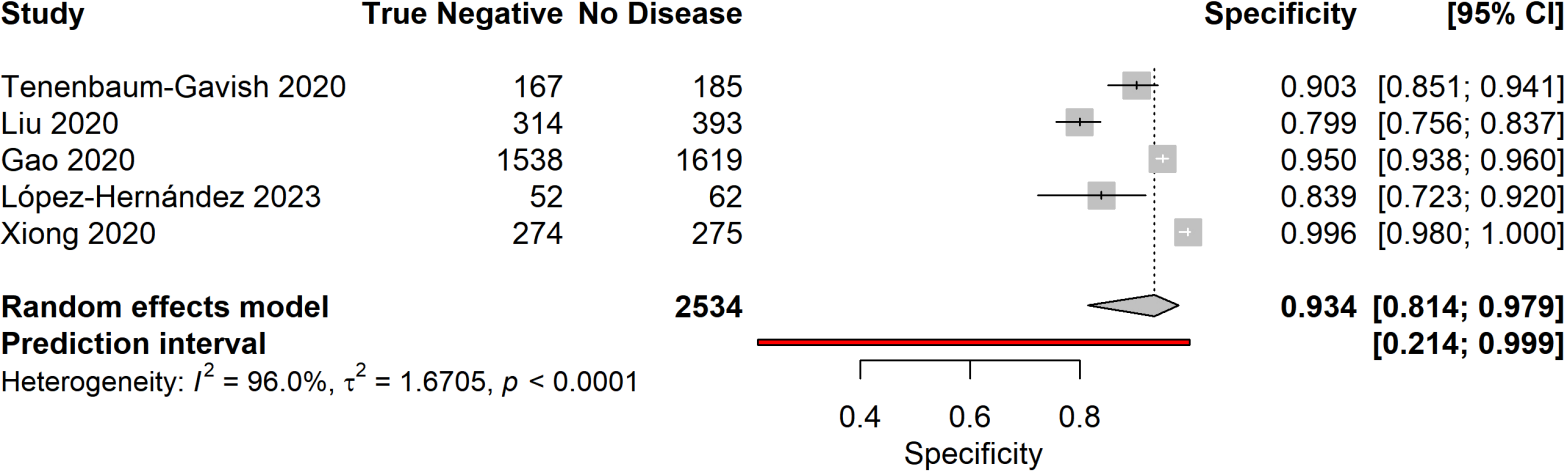
Forest plot of pooled specificity for early pregnancy biomarkers predicting GDM. Random effects model yielded pooled specificity of 93.4% (95% CI: 81.4%-97.9%) with substantial heterogeneity (I²=96.0%).

**Figure 6:**
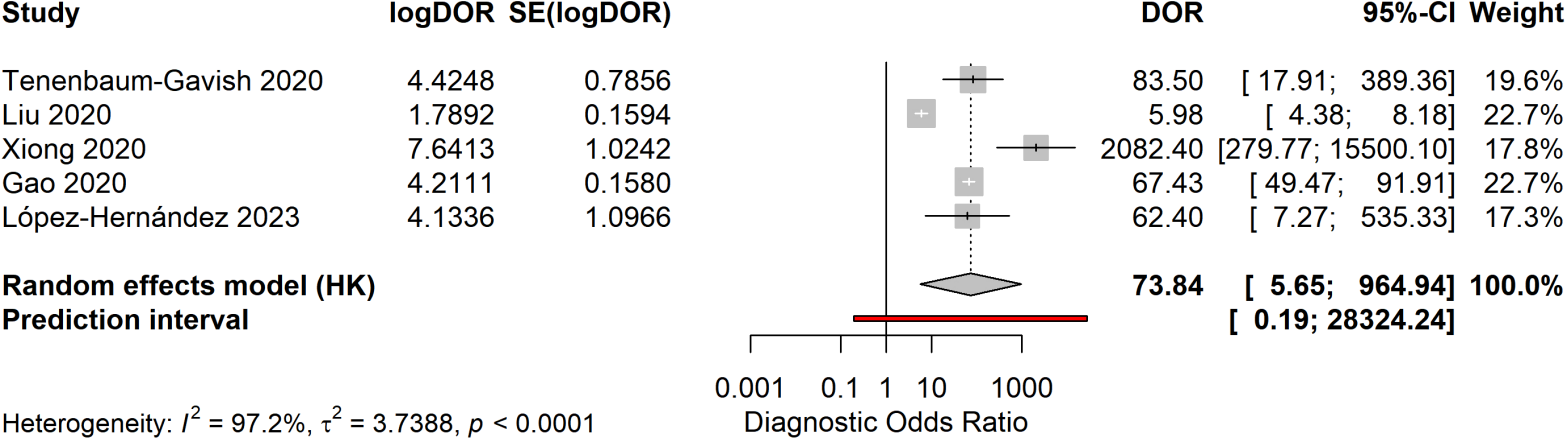
Forest plot of diagnostic odds ratios for early pregnancy biomarkers. Pooled DOR of 73.84 (95% CI: 5.65-964.94) indicates good discriminative ability, though wide confidence interval reflects substantial heterogeneity (I²=97.2%).

**Figure 7:**
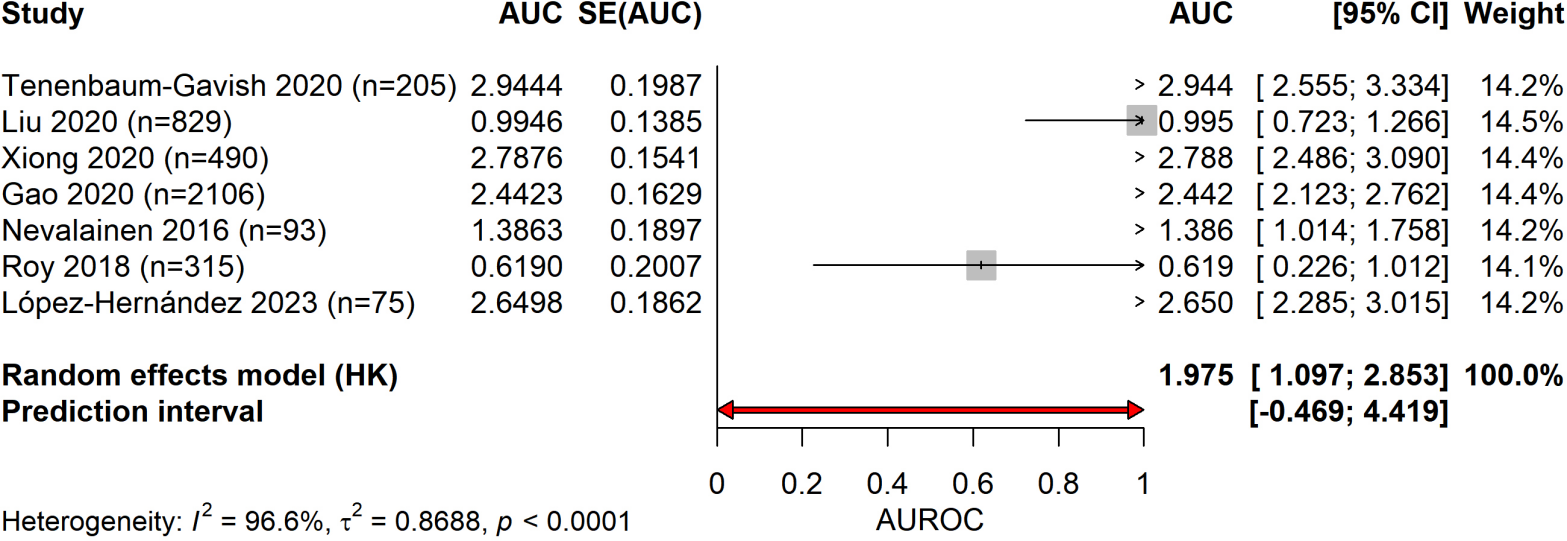
Forest plot of AUROC values for early pregnancy biomarkers. Studies demonstrated AUROC values ranging from 0.619 to 2.944 (values >1 represent transformed estimates), with pooled estimate of 1.975 (95% CI: 1.097-2.853).

**Figure 8:**
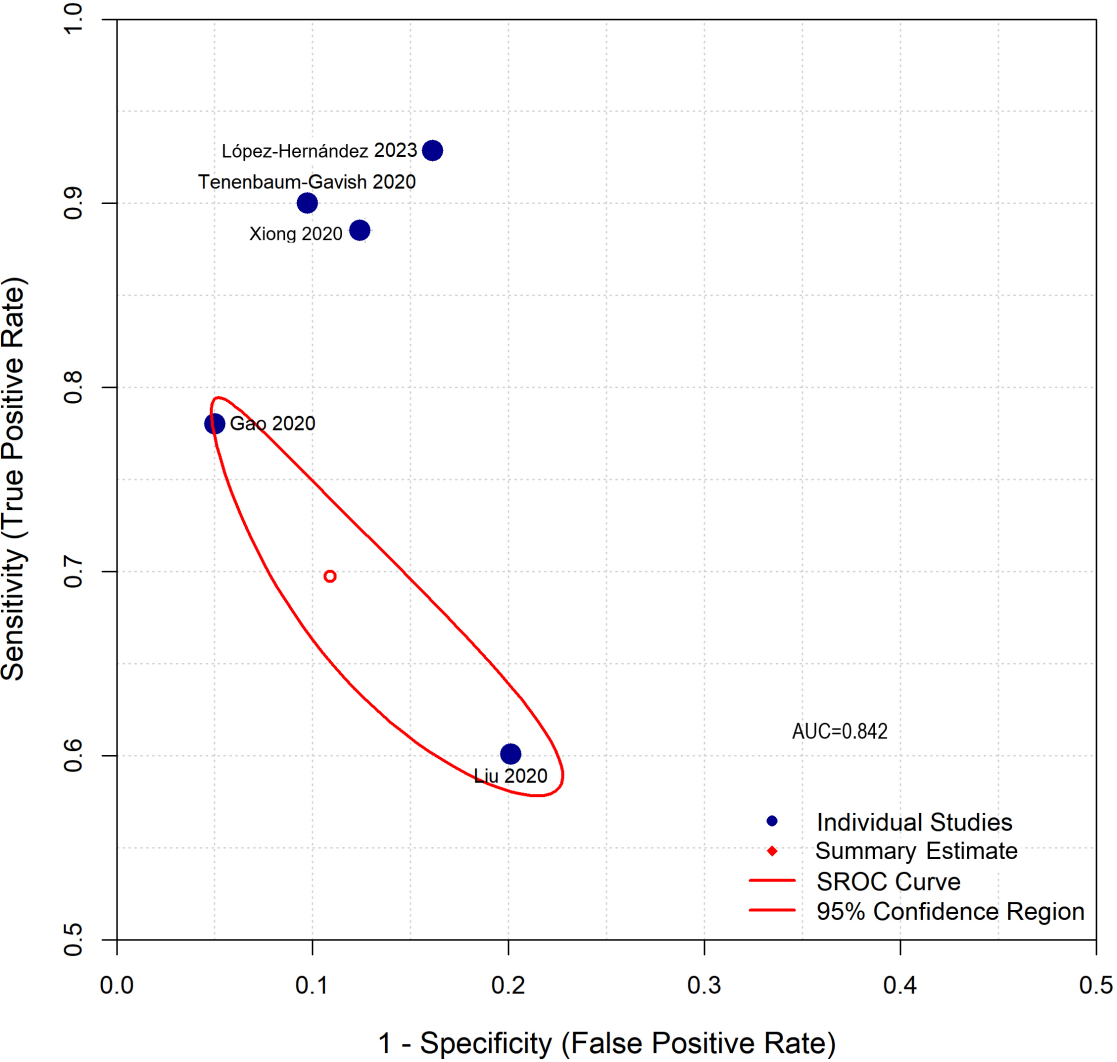
Summary receiver operating characteristic (SROC) curve. The curve shows good overall discriminative ability with summary AUROC of 0.881 (shown as AUC = 0.842 in the curve due to different calculation method), with wide 95% confidence region reflecting substantial between-study heterogeneity.

Meta-analysis of five studies reporting complete diagnostic accuracy data yielded pooled sensitivity of 81.6% (95% CI: 68.7%-90.0%), pooled specificity of 93.4% (95% CI: 81.4%-97.9%), and pooled diagnostic odds ratio of 73.84 (95% CI: 5.65-964.94) (**Figures 4**–**6**). The summary AUROC was 0.881 (95% CI: 0.795-0.967), indicating good overall discriminative ability (**Figures 7**, **8**). However, substantial heterogeneity was present across all metrics (I²=94.3-97.2%, all p<0.0001), reflecting variations in biomarker types, study populations, GDM diagnostic criteria, and analytical approaches. The prediction intervals for sensitivity (34.7%-97.4%) and specificity (21.4%-99.9%) were wide, indicating considerable variability in diagnostic performance across different settings and populations.

### Publication bias and sensitivity analysis

**Figure 9:**
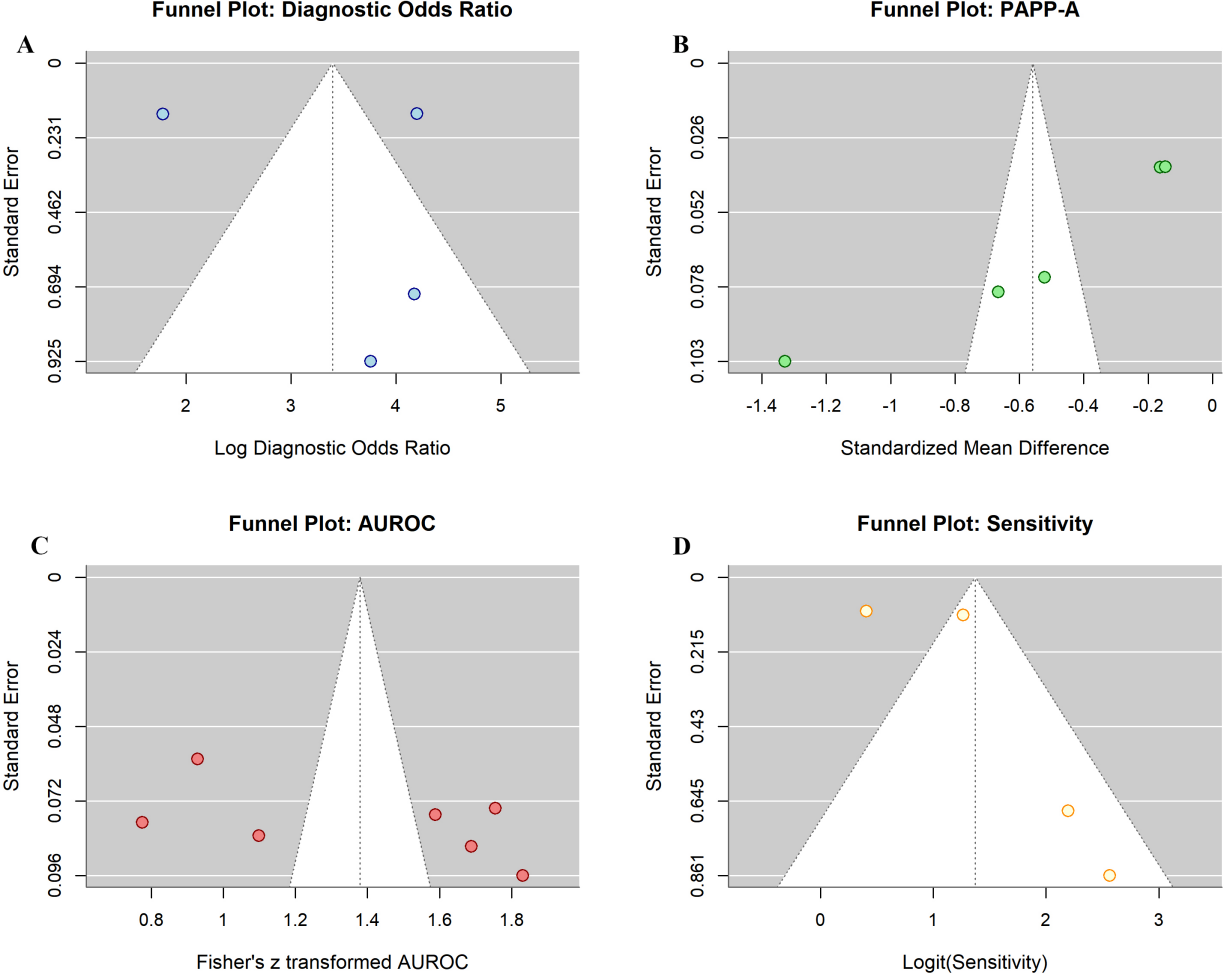
Funnel plots assessing publication bias for **(A)** diagnostic odds ratio, **(B)** PAPP-A standardized mean difference, **(C)** AUROC values, and **(D)** sensitivity. Reasonable symmetry across all plots suggests minimal publication bias. Egger’s test was not statistically significant (all p>0.10).

Visual inspection of funnel plots for diagnostic odds ratio, PAPP-A standardized mean difference, AUROC, and sensitivity showed reasonable symmetry, suggesting minimal publication bias (**Figure 9**). Egger’s regression test was not statistically significant for any outcome (all p>0.10), providing no evidence of small-study effects. Sensitivity analyses sequentially excluding individual studies did not substantially alter pooled estimates or reduce heterogeneity, indicating that results were not driven by any single influential study. The high heterogeneity appears to reflect genuine differences in biomarker types, measurement methods, and population characteristics rather than publication bias or individual study outliers.

## Discussion

This systematic review and meta-analysis evaluated the diagnostic accuracy of early pregnancy biomarkers for GDM prediction, synthesizing evidence from 12 studies encompassing 79, 233 participants. Our findings indicate that early pregnancy biomarkers show moderate to high diagnostic accuracy, with pooled sensitivity of 81.6%, specificity of 93.4%, diagnostic odds ratio of 73.84, and AUROC of 0.881. Metabolomic markers, particularly acylcarnitines C5 and C5:1, and machine learning models combining multiple clinical and biochemical parameters demonstrated the best performance. However, it is crucial to interpret these exceptional results with caution. Some high AUROC values were derived from relatively small cohorts, which carries a significant risk of model overfitting. Therefore, these promising findings must be considered preliminary and require rigorous validation in larger, independent populations.

The pathophysiological basis for early biomarker alterations in women who subsequently develop GDM is increasingly well understood. Acylcarnitines, intermediates in fatty acid oxidation, are elevated early in pregnancy in women destined to develop GDM, reflecting mitochondrial dysfunction and altered substrate metabolism that precede overt glucose intolerance (42, 43). Similarly, inflammatory cytokines such as TNF-α and IL-6 disrupt insulin signaling pathways and contribute to pregnancy-induced insulin resistance (44–46). PAPP-A, which regulates IGF bioavailability and placental function, may reflect early placental dysfunction associated with metabolic dysregulation. These mechanistic insights support the biological plausibility of early biomarker-based prediction and suggest potential therapeutic targets for GDM prevention.

Our meta-analysis of PAPP-A levels revealed a non-significant trend toward lower values in women who developed GDM (SMD -0.558, p=0.067). While individual studies have reported statistically significant associations (32, 33), the pooled estimate did not reach significance, possibly reflecting the modest effect size and substantial between-study heterogeneity. The clinical utility of PAPP-A alone for GDM prediction appears limited, though it may contribute to multivariate prediction models combining multiple first-trimester markers.

The machine learning models evaluated in included studies showed promising performance, with several achieving AUROC values exceeding 0.90. However, important methodological concerns limit confidence in these results. Most studies used internal validation methods (split-sample or cross-validation) rather than external validation in independent cohorts, which typically yields overly optimistic performance estimates (47). Furthermore, the specific features and algorithms varied substantially across studies, making it difficult to identify optimal model specifications. The integration of ensemble methods and explainable AI techniques represents a promising direction for improving prediction accuracy while maintaining clinical interpretability (30–32). Future studies should prioritize external validation in diverse populations and transparent reporting of model development and validation procedures following established guidelines such as TRIPOD (Transparent Reporting of a multivariable prediction model for Individual Prognosis Or Diagnosis) (48).

The single study evaluating inflammatory markers in a comprehensive manner (Tenenbaum-Gavish 2020) reported excellent performance for a TNF-α-based prediction model, aligning with biological understanding of inflammation’s role in GDM pathogenesis. However, as this represents findings from only one study, replication in independent cohorts is essential before drawing firm conclusions about inflammatory markers’ clinical utility. The paucity of studies examining inflammatory markers is surprising given their well-established mechanistic links to insulin resistance and GDM (44–46), highlighting a gap in the current literature.

Metabolomic approaches identifying specific acylcarnitine species appear particularly promising, with multiple studies demonstrating strong associations and good discrimination (49–51). The consistency of findings across different populations and the clear biological rationale strengthen confidence in these biomarkers. However, metabolomic assays require specialized equipment and expertise, potentially limiting their widespread clinical implementation. Cost-effectiveness analyses comparing metabolomic screening strategies with current standard care are needed to inform policy decisions (33–36).

The practical implementation of early biomarker screening raises several considerations. The optimal screening strategy likely involves a staged approach, beginning with readily available clinical and biochemical information (maternal age, BMI, family history, fasting glucose) to identify higher-risk women who would benefit from more specialized biomarker testing. Economic evaluations have suggested that such risk-stratified approaches may be more cost-effective than universal specialized biomarker screening (35, 36). Moreover, early identification of high-risk women must be coupled with evidence-based interventions to prevent or delay GDM onset. Lifestyle modification programs initiated in early pregnancy have demonstrated modest benefits in reducing GDM incidence (48, 52), though optimal intervention timing, intensity, and components remain areas of active investigation.

Strengths of our meta-analysis include comprehensive search strategy, rigorous quality assessment, appropriate statistical methods accounting for diagnostic test accuracy characteristics, and inclusion of diverse biomarker types. We provide detailed sample size considerations specific to diagnostic meta-analyses, report search dates and database coverage precisely, include findings from ClinicalTrials.gov registry searches, and acknowledge limitations regarding sparse data on integrated multi-marker panels. However, several limitations warrant consideration. The substantial heterogeneity across studies, reflected in I² values exceeding 94% for most outcomes, limits the precision and generalizability of pooled estimates. This heterogeneity likely stems from differences in biomarker types, assay methods, GDM diagnostic criteria, population characteristics, and analytical approaches. We were unable to comprehensively explore sources of heterogeneity through subgroup analyses due to limited study numbers for most biomarker categories. Publication bias, while not detected by statistical tests, remains a possibility as studies with null findings may be less likely to be published. Most included studies had relatively small sample sizes and were conducted in single centers, potentially limiting generalizability. The lack of standardized reporting of machine learning model development and validation procedures hampered our ability to fully evaluate these approaches’ reliability.

Future research should prioritize several key areas. Large-scale, prospective cohort studies with standardized biomarker measurement protocols and GDM diagnostic criteria are needed to reduce heterogeneity and provide more precise effect estimates. External validation of promising biomarker panels and prediction models in diverse populations is essential to assess generalizability and inform clinical implementation. Economic analyses examining cost-effectiveness of various screening strategies across different healthcare systems and populations would help guide policy decisions (35, 36). Integration of emerging artificial intelligence and machine learning techniques with explainable AI frameworks (30–32) may improve prediction accuracy while maintaining clinical interpretability. Mechanistic studies investigating the causal relationships between early biomarker alterations and GDM development could identify novel therapeutic targets for prevention. Finally, intervention trials testing whether early identification and targeted prevention strategies can reduce GDM incidence and improve maternal-fetal outcomes are needed to establish clinical utility beyond prognostic value.

In conclusion, early pregnancy biomarkers demonstrate moderate to high diagnostic accuracy for GDM prediction, with metabolomic markers and integrated multi-parameter models showing particular promise based on available data. However, high heterogeneity across studies, methodological variations, and limited data on integrated approaches constrain immediate clinical application. Standardization of measurement protocols, external validation in diverse populations, and rigorous cost-effectiveness analyses are necessary steps before widespread clinical implementation. When these conditions are met, early biomarker-based screening could enable risk stratification and targeted interventions that improve outcomes for mothers and infants affected by GDM.

## Data Availability

The original contributions presented in the study are included in the article/**Supplementary Material**. Further inquiries can be directed to the corresponding author.

## References

[1] International Diabetes Federation . IDF Diabetes Atlas. 10th ed. Brussels, Belgium: International Diabetes Federation (2021).

[2] Saeedi P Petersohn I Salpea P Malanda B Karuranga S Unwin N . Global and regional diabetes prevalence estimates for 2019 and projections for 2030 and 2045: Results from the International Diabetes Federation Diabetes Atlas, 9th edition. Diabetes Res Clin Pract. (2019) 157:107843. doi: 10.1016/j.diabres.2019.107843, PMID: 31518657

[3] HAPO Study Cooperative Research Group . Hyperglycemia and Adverse Pregnancy Outcome (HAPO) Study: associations with neonatal anthropometrics. Diabetes. (2009) 58:453–9. doi: 10.2337/db08-1112, PMID: 19011170 PMC2628620

[4] Bellamy L Casas JP Hingorani AD Williams D . Type 2 diabetes mellitus after gestational diabetes: a systematic review and meta-analysis. Lancet. (2009) 373:1773–9. doi: 10.1016/S0140-6736(09)60731-5, PMID: 19465232

[5] Damm P Houshmand-Oeregaard A Kelstrup L Lauenborg J Mathiesen ER Clausen TD . Gestational diabetes mellitus and long-term consequences for mother and offspring: a view from Denmark. Diabetologia. (2016) 59:1396–9. doi: 10.1007/s00125-016-3985-5, PMID: 27174368

[6] American College of Obstetricians and Gynecologists . ACOG Practice Bulletin No. 190: Gestational diabetes mellitus. Obstet Gynecol. (2018) 131:e49–64. 10.1097/AOG.0000000000002501 29370047

[7] Sacks DB . Diagnosis of gestational diabetes mellitus: it is time for international consensus. Clin Chem. (2014) 60:141–3. doi: 10.1373/clinchem.2013.206920, PMID: 24061614

[8] Lowe WL Jr Scholtens DM Kuang A Linder B Lawrence JM Lebenthal Y . Hyperglycemia and Adverse Pregnancy Outcome Follow-up Study (HAPO FUS): maternal gestational diabetes mellitus and childhood glucose metabolism. Diabetes Care. (2019) 42:372–80. doi: 10.2337/dc18-1646, PMID: 30655380 PMC6385693

[9] Powe CE Allard C Battista MC Doyon M Bouchard L Ecker JL . Heterogeneous contribution of insulin sensitivity and secretion defects to gestational diabetes mellitus. Diabetes Care. (2016) 39:1052–5. doi: 10.2337/dc15-2672, PMID: 27208340 PMC4878218

[10] Catalano PM Huston L Amini SB Kalhan SC . Longitudinal changes in glucose metabolism during pregnancy in obese women with normal glucose tolerance and gestational diabetes mellitus. Am J Obstet Gynecol. (1999) 180:903–16. doi: 10.1016/S0002-9378(99)70662-9, PMID: 10203659

[11] Plows JF Stanley JL Baker PN Reynolds CM Vickers MK . The pathophysiology of gestational diabetes mellitus. Int J Mol Sci. (2018) 19:3342. doi: 10.3390/ijms19113342, PMID: 30373146 PMC6274679

[12] Pantham P Aye IL Powell TL . Inflammation in maternal obesity and gestational diabetes mellitus. Placenta. (2015) 36:709–15. doi: 10.1016/j.placenta.2015.04.006, PMID: 25972077 PMC4466145

[13] Lekva T Norwitz ER Aukrust P Ueland T . Impact of systemic inflammation on the progression of gestational diabetes mellitus. Curr Diabetes Rep. (2016) 16:26. doi: 10.1007/s11892-016-0715-9, PMID: 26879309

[14] Shao J Catalano PM Yamashita H Ruyter I Smith S Youngren J . Decreased insulin receptor tyrosine kinase activity and plasma cell membrane glycoprotein-1 overexpression in skeletal muscle from obese women with gestational diabetes mellitus (GDM): evidence for increased serine/threonine phosphorylation in pregnancy and GDM. Diabetes. (2000) 49:603–10. doi: 10.2337/diabetes.49.4.603, PMID: 10871198

[15] Hotamisligil GS Peraldi P Budavari A Ellis R White MF Spiegelman BM . IRS-1-mediated inhibition of insulin receptor tyrosine kinase activity in TNF-alpha- and obesity-induced insulin resistance. Science. (1996) 271:665–8. doi: 10.1126/science.271.5249.665, PMID: 8571133

[16] Pradhan AD Manson JE Rifai N Buring JE Ridker PM . C-reactive protein, interleukin 6, and risk of developing type 2 diabetes mellitus. JAMA. (2001) 286:327–34. doi: 10.1001/jama.286.3.327, PMID: 11466099

[17] Kirwan JP Hauguel-De Mouzon S Lepercq J Challier JC Huston-Presley L Friedman JE . TNF-alpha is a predictor of insulin resistance in human pregnancy. Diabetes. (2002) 51:2207–13. doi: 10.2337/diabetes.51.7.2207, PMID: 12086951

[18] Beneventi F Simonetta M Lovati E Albonico G Tinelli C Locatelli E . First trimester pregnancy-associated plasma protein-A in pregnancies complicated by subsequent gestational diabetes. Prenat Diagn. (2011) 31:523–8. doi: 10.1002/pd.2733, PMID: 21404306

[19] Nicolaides KH Syngelaki A Ashoor G Birdir C Touzet G . Noninvasive prenatal testing for fetal trisomies in a routinely screened first-trimester population. Am J Obstet Gynecol. (2012) 207:374.e1–6. doi: 10.1016/j.ajog.2012.08.033, PMID: 23107079

[20] Dugoff L Hobbins JC Malone FD Porter TF Luthy D Comstock CH . First-trimester maternal serum PAPP-A and free-beta subunit human chorionic gonadotropin concentrations and nuchal translucency are associated with obstetric complications: a population-based screening study (the FASTER Trial). Am J Obstet Gynecol. (2004) 191:1446–51. doi: 10.1016/j.ajog.2004.06.052, PMID: 15507981

[21] Enquobahrie DA Williams MA Qiu C Meller M Sorensen TK . Global placental gene expression in gestational diabetes mellitus. Am J Obstet Gynecol. (2009) 200:206.e1–13. doi: 10.1016/j.ajog.2008.08.022, PMID: 18845290

[22] Pappa KI Vlachos G Theodora M Roubelaki M Angelidou K Antsaklis A . Intermediate metabolism in association with the amino acid profile during the third trimester of normal pregnancy and diet-controlled gestational diabetes. Am J Obstet Gynecol. (2007) 196:65.e1–5. doi: 10.1016/j.ajog.2006.06.094, PMID: 17240238

[23] Scholtens DM Bain JR Reisetter AC Muehlbauer MJ Nodzenski M Stevens RD . Metabolic networks and metabolites underlie associations between maternal glucose during pregnancy and newborn size at birth. Diabetes. (2016) 65:2039–50. doi: 10.2337/db15-1748, PMID: 27207545 PMC4915585

[24] Lowe WL Jr Bain JR Nodzenski M Reisetter AC Muehlbauer MJ Stevens RD . Maternal BMI and glycemia impact the fetal metabolome. Diabetes Care. (2017) 40:902–10. doi: 10.2337/dc16-2452, PMID: 28637888 PMC5481987

[25] International Association of Diabetes and Pregnancy Study Groups Consensus Panel . International association of diabetes and pregnancy study groups recommendations on the diagnosis and classification of hyperglycemia in pregnancy. Diabetes Care. (2010) 33:676–82. doi: 10.2337/dc09-1848, PMID: 20190296 PMC2827530

[26] American Diabetes Association . Classification and diagnosis of diabetes: standards of medical care in diabetes-2020. Diabetes Care. (2020) 43:S14–31. doi: 10.2337/dc20-S002, PMID: 31862745

[27] Lamain-de Ruiter M Kwee A Naaktgeboren CA Groot ID Evers IM Groenendaal F . External validation of prognostic models to predict risk of gestational diabetes mellitus in one Dutch cohort: prospective multicentre cohort study. BMJ. (2016) 354:i4338. doi: 10.1136/bmj.i4338, PMID: 27576867

[28] Sweeting A Park F Hyett J . The first trimester: prediction and prevention of the great obstetrical syndromes. Best Pract Res Clin Obstet Gynaecol. (2015) 29:183–93. doi: 10.1016/j.bpobgyn.2014.09.006, PMID: 25482532

[29] Sufriyana H Husnayain A Chen YW Kuo CY Singh O Yeh TY . Comparison of multivariable logistic regression and other machine learning algorithms for prognostic prediction studies in pregnancy care: systematic review and meta-analysis. JMIR Med Inform. (2020) 8:e16503. doi: 10.2196/16503, PMID: 33200995 PMC7708089

[30] Hassan A Ahmed A . Predicting Parkinson’s disease progression: a noninvasive method leveraging voice inputs. Comput Sci. (2023) 8:66–82. doi: 10.53070/bbd.1350356

[31] Ahmad SG Arif MA Hassan A Ayyub K Munir EU Ramzan N . IoT-based smart wearable belt for tracking fetal kicks and movements in expectant mothers. IEEE Sens J. (2025) 27322–27333.

[32] Hassan A Nawaz S Tahira S Ahmad A . Preterm birth prediction using an explainable machine learning approach. Artif Intell Appl. (2025). doi: 10.47852/bonviewAIA52024517

[33] Werner EF Pettker CM Zuckerwise L Reel M Funai EF Henderson J . Screening for gestational diabetes mellitus: are the criteria proposed by the International Association of the Diabetes and Pregnancy Study Groups cost-effective? Diabetes Care. (2012) 35:529–35. doi: 10.2337/dc11-1643, PMID: 22266735 PMC3322683

[34] Mission JF Ohno MS Cheng YW Caughey AB . Gestational diabetes screening with the new IADPSG guidelines: a cost-effectiveness analysis. Am J Obstet Gynecol. (2012) 207:326.e1–9. doi: 10.1016/j.ajog.2012.06.048, PMID: 22840972 PMC4621259

[35] Hassan A Ahmad SG Iqbal T Munir EU Ayyub K Ramzan N . Enhanced model for gestational diabetes mellitus prediction using a fusion technique of multiple algorithms with explainability. Int J Comput Intell Syst. (2025) 18:1–33. doi: 10.1007/s44196-025-00760-4, PMID: 41868966

[36] Marseille E Lohse N Jiwani A Hod M Seshiah V Yajnik CS . The cost-effectiveness of gestational diabetes screening including prevention: application of a new model in India and Israel. J Matern Fetal Neonatal Med. (2013) 26:802–10. doi: 10.3109/14767058.2013.765845, PMID: 23311860

[37] Page MJ McKenzie JE Bossuyt PM Boutron I Hoffmann TC Mulrow CD . The PRISMA 2020 statement: an updated guideline for reporting systematic reviews. BMJ. (2021) 372:n71. 33782057 10.1136/bmj.n71 PMC8005924

[38] Whiting PF Rutjes AWS Westwood ME Mallett S Deeks JJ Reitsma JB . QUADAS-2: a revised tool for the quality assessment of diagnostic accuracy studies. Ann Intern Med. (2011) 155:529–36. doi: 10.7326/0003-4819-155-8-201110180-00009, PMID: 22007046

[39] Riley RD Higgins JPT Deeks JJ . Interpretation of random effects meta-analyses. BMJ. (2011) 342:d549. doi: 10.1136/bmj.d549, PMID: 21310794

[40] Cochrane Handbook for Systematic Reviews of Diagnostic Test Accuracy. Version 2.0 (2025). Available online at: https://methods.cochrane.org/sdt/handbook-dta-reviews (Accessed January 10, 2025).

[41] Reitsma JB Glas AS Rutjes AW Scholten Rob JPM Bossuyt PM Zwinderman AH . Bivariate analysis of sensitivity and specificity produces informative summary measures in diagnostic reviews. J Clin Epidemiol. (2005) 58:982–90. doi: 10.1016/j.jclinepi.2005.02.022, PMID: 16168343

[42] Lovati E Beneventi F Simonetta M Laneri M Quarleri L Scudeller L . Gestational diabetes mellitus: including serum pregnancy-associated plasma protein-A testing in the clinical management of primiparous women? A case-control study. Diabetes Res Clin Pract. (2013) 100:340–7. doi: 10.1016/j.diabres.2013.04.002, PMID: 23642968

[43] López-Hernández Y Herrera-Van Oostdam AS Toro-Ortíz JC López JA Salgado-Bustamante M Murgu M . Urinary metabolites altered during the third trimester in pregnancies complicated by gestational diabetes mellitus: relationship with potential upcoming metabolic disorders. Int J Mol Sci. (2019) 20:1186. doi: 10.3390/ijms20051186, PMID: 30857174 PMC6429483

[44] Chu YL Gong YD Su ZH Yu 1HN Cui Q Jiang HY . Relationship between tyrosine phosphorylation and protein expression of insulin receptor and insulin resistance in gestational diabetes mellitus. J Huazhong Univ Sci Technol Med Sci. (2014) 34:393–7. doi: 10.1007/s11596-014-1289-x, PMID: 24939305

[45] Atègbo JM Grissa O Yessoufou A . Modulation of adipokines and cytokines in gestational diabetes and macrosomia. J Clin Endocrinol Metab. (2006) 91:4137–43. doi: 10.1210/jc.2006-0980, PMID: 16849405

[46] Siddiqui S Waghdhare S Goel C Panda M Soneja H Sundar J . Augmentation of IL-6 production contributes to development of gestational diabetes mellitus: an Indian study. Diabetes Metab Syndr Clin Res Rev. (2019) 13:895–9. doi: 10.1016/j.dsx.2018.12.023, PMID: 31336542

[47] Ye Y Xiong Y Zhou Q Wu JN Li XT Xiao XR . Comparison of machine learning methods and conventional logistic regressions for predicting gestational diabetes using routine clinical data: a retrospective cohort study. J Diabetes Res. (2020) 2020:4168340. doi: 10.1155/2020/4168340, PMID: 32626780 PMC7306091

[48] Zakaria H Abusanana S Mussa BM Dhaheri ASA Stojanovska L Mohamad MN . The role of lifestyle interventions in the prevention and treatment of gestational diabetes mellitus. Med (Kaunas). (2023) 59:287. doi: 10.3390/medicina59020287, PMID: 36837488 PMC9966224

[49] Roy C Tremblay PY Anassour-Laouan-Sidi E Lucas M Forest JC Giguère Y . Risk of gestational diabetes mellitus in relation to plasma concentrations of amino acids and acylcarnitines: a nested case-control study. Diabetes Res Clin Pract. (2018) 140:183–90. doi: 10.1016/j.diabres.2018.03.058, PMID: 29626588

[50] Nevalainen J Sairanen M Appelblom H Gissler M Timonen S Ryynänen M . First-trimester maternal serum amino acids and acylcarnitines are significant predictors of gestational diabetes. Rev Diabetes Stud. (2016) 13:236–45. doi: 10.1900/RDS.2016.13.236, PMID: 28278310 PMC5734224

[51] Batchuluun B Al Rijjal D Prentice KJ Eversley JA Burdett E Mohan H . Elevated medium-chain acylcarnitines are associated with gestational diabetes mellitus and early progression to type 2 diabetes and induce pancreatic beta-cell dysfunction. Diabetes. (2018) 67:885–97. doi: 10.2337/db17-1150, PMID: 29436377 PMC5910003

[52] Tsironikos GI Potamianos P Zakynthinos GE Tsolaki V Tatsioni A Bargiota A . Effectiveness of lifestyle interventions during pregnancy on preventing gestational diabetes mellitus in high-risk women: a systematic review and meta-analyses of published RCTs. J Clin Med. (2023) 12:7038. doi: 10.3390/jcm12227038, PMID: 38002654 PMC10672732

