# Supplementary material for: Early pregnancy biomarkers for gestational diabetes mellitus prediction: a systematic review and meta-analysis of routine laboratory, metabolic, and inflammatory markers
Source: Front Endocrinol (Lausanne). 2026 Mar 26;17:1749694. doi: 10.3389/fendo.2026.1749694 (PMC13061706; doi:10.3389/fendo.2026.1749694)
Supplement: Supplementary file 1 [file Table1.docx]

**Supplementary Materials**

**Supplementary Table 1. Characteristics of included studies including author, year, country, study design, sample size, participant demographics (age, BMI, ethnicity), biomarkers evaluated, measurement timing and assay methods, GDM diagnostic criteria, and key findings.**

| Study (first author, year) | Country | Study design | Sample size (GDM / non-GDM) | Participant demographics (age, BMI, ethnicity)* | Biomarkers evaluated | Measurement timing | Assay methods* | GDM diagnostic criteria | Key findings (summary, based on current manuscript) |
| --- | --- | --- | --- | --- | --- | --- | --- | --- | --- |
| Tenenbaum-Gavish 2020 [42] | Israel | Prospective cohort | 20 / 185 | NR in current manuscript | TNF-α plus clinical factors (age, BMI, family history) and routine biochemistry (glucose, lipids) | First trimester (≈11–13+6 weeks) | NR in main text; reported as standard serum cytokine and biochemical assays in original study | IADPSG | Early-pregnancy model combining TNF-α, maternal factors and routine labs showed high apparent performance (AUC ≈0.95; Se 0.90, Sp 0.90) in a small cohort. |
| Beneventi 2011 [18] | Italy | Case-control | 228 / 228 | NR | PAPP-A, free β-hCG | First trimester | NR; PAPP-A expressed as MoM from routine aneuploidy screening | Carpenter–Coustan | First-trimester PAPP-A levels were lower in women who later developed GDM (0.7 vs 1.2 MoM); no formal diagnostic accuracy metrics reported. |
| Lovati 2013 [43] | Italy | Case-control | 307 / 366 | NR | PAPP-A, free β-hCG | First trimester | NR; PAPP-A reported as MoM | IADPSG / ADA | PAPP-A significantly lower in GDM group (0.9±0.6 vs 1.3±0.6 MoM); GDM associated with low PAPP-A but predictive value alone appeared limited. |
| Savvidou 2012 [44] | UK | Cohort | 779 / 41,007 | NR | PAPP-A, free β-hCG | 11–13+6 weeks | NR; first-trimester combined screening assay (MoM values) | WHO | Large screening cohort; women who later developed GDM had slightly lower PAPP-A (0.94 vs 1.00 MoM); effect small and not sufficient for standalone screening. |
| Syngelaki 2015 [45] | UK | Cohort | 787 / 30,438 | NR | PAPP-A | 11–13+6 weeks | NR; PAPP-A MoM from routine first-trimester screening | WHO 1999 | PAPP-A modestly lower in those who developed GDM (0.949 vs 1.000 MoM); authors concluded PAPP-A alone has limited predictive utility. |
| Sweeting 2017 [46] | Australia | Case-control | 248 / 732 | NR | PAPP-A, free β-hCG | 11–13+6 weeks | NR; routine first-trimester serum screening | ADIPS | Lower first-trimester PAPP-A in GDM group (0.81 vs 1.00 MoM); addition of PAPP-A to clinical risk factors only modestly improved discrimination. |
| Liu 2020 [47] | China | Prospective cohort | 436 / 393 | NR; routine obstetric population | Fasting plasma glucose (FPG) + age + BMI (machine-learning model) | <19 weeks (early pregnancy) | NR; routine laboratory FPG and clinical data | IADPSG | Machine-learning model using FPG, age and BMI showed moderate accuracy (Se 0.601, Sp 0.799, AUC 0.73); external validation not reported. |
| Xiong 2020 [48] | China | Retrospective cohort | 490 / NA | NR | Routine coagulation parameters (PT, APTT) used in a combined model | <19 weeks | NR; routine coagulation assays on hospital analyzers | ADA | Early-pregnancy model based on PT and APTT achieved high apparent accuracy (Se 0.883, Sp 0.995, AUC 0.942); single-center data; needs external validation. |
| Gao 2020 [49] | China | Prospective cohort | 487 / 1,619 | NR; report indicates higher age/BMI and family history in GDM group | Maternal age, family history of diabetes (FHD), BMI (clinical risk score) | First trimester | NR; clinical risk factors only (no novel assay) | IADPSG | Clinical risk score based on age, BMI and FHD showed good discrimination (Se 0.780, Sp 0.950, AUC 0.920) for GDM; developed and tested in a single cohort. |
| Nevalainen 2016 [50] | Finland | Prospective cohort | 31 / 62 | NR | Targeted metabolomics: acylcarnitines + amino acids | First trimester | NR in main text; original paper reports targeted MS-based metabolomics | Finnish national criteria | Small metabolomics study; panels of acylcarnitines/amino acids provided moderate discrimination (AUC ~0.77–0.83); risk of overfitting due to small sample. |
| Roy 2018 [51] | Canada | Nested case-control | 162 / 153 | NR | Serum acylcarnitines | 11–14 weeks | NR in main text; targeted MS-based metabolomic profiling | Canadian diagnostic criteria | Early-pregnancy acylcarnitine profiles associated with later GDM, but overall AUROC was modest (≈0.65); no single metabolite had strong standalone value. |
| López-Hernández 2023 [52] | Mexico | Prospective cohort | 13 / 62 | NR | Short-chain acylcarnitines C5 and C5:1 plus clinical factors | <18 weeks | NR; LC-MS measurement of C5/C5:1 reported in original paper | IADPSG | Very small pilot cohort; C5/C5:1-based model showed high apparent accuracy (Se 0.929, Sp 0.839, AUC 0.934); wide uncertainty, requires independent validation. |
